# Performance and grain yield stability of maize populations developed using marker-assisted recurrent selection and pedigree selection procedures

**DOI:** 10.1007/s10681-015-1590-1

**Published:** 2015-11-09

**Authors:** Yoseph Beyene, Kassa Semagn, Stephen Mugo, Boddupalli M. Prasanna, Amsal Tarekegne, John Gakunga, Pierre Sehabiague, Barbara Meisel, Sylvester O. Oikeh, Michael Olsen, Jose Crossa

**Affiliations:** International Maize and Wheat Improvement Center (CIMMYT), P.O. Box 1041, Village Market, Nairobi, 00621 Kenya; CIMMYT, 12.5 km peg Mazowe Road, Mount Pleasant, P.O. Box MP163, Harare, Zimbabwe; Monsanto SAS, Croix de Pardies, BP21, 40305 Peyrehorade, France; Monsanto South Africa (Pty) Ltd, P.O. Box 69933, Bryanston, 2021 South Africa; African Agricultural Technology Foundation (AATF), P.O. Box 30709-00100, Nairobi, Kenya; International Maize and Wheat Improvement Center (CIMMYT), Apdo. Postal 6-641, 06600 Mexico, DF Mexico

**Keywords:** Africa, Drought, Molecular breeding, SNP, Rapid cycle recombination, Testcross evaluation

## Abstract

A marker-assisted recurrent selection (MARS) program was undertaken in sub-Saharan Africa to improve grain yield under drought-stress in 10 biparental tropical maize populations. The objectives of the present study were to evaluate the performance of C_1_S_2_-derived hybrids obtained after three MARS cycles (one cycle of recombination (C_1_), followed by two generations of selfing (S_2_), and to study yield stability under both drought-stress (DS) and well-watered (WW) conditions. For each of the 10 populations, we evaluated hybrids developed by crossing 47–74 C_1_S_2_ lines advanced through MARS, the best five S_5_ lines developed through pedigree selection, and the founder parents with a single-cross tester from a complementary heterotic group. The hybrids and five commercial checks were evaluated in Kenya under 1–3 DS and 3–5 WW conditions with two replications. Combined across DS locations, the top 10 C_1_S_2_-derived hybrids from each of the 10 biparental populations produced 0.5–46.3 and 11.1–55.1 % higher mean grain yields than hybrids developed using pedigree selection and the commercial checks, respectively. Across WW locations, the best 10 hybrids derived from C_1_S_2_ of each population produced 3.4–13.3 and 7.9–36.5 % higher grain yields than hybrids derived using conventional pedigree breeding and the commercial checks, respectively. Mean days to anthesis of the best 10 C_1_S_2_ hybrids were comparable to those of hybrids developed using the pedigree method, the founder parents and the commercial checks, with a maximum difference of 3.5 days among the different groups. However, plant height was significantly (P < 0.01) different in most pairwise comparisons. Our results showed the superiority of MARS over pedigree selection for improving diverse tropical maize populations as sources of improved lines for stress-prone environments and thus MARS can be effectively integrated into mainstream maize breeding programs.

**Electronic supplementary material:**

The online version of this article (doi:10.1007/s10681-015-1590-1) contains supplementary material, which is available to authorized users.

## Introduction

Maize, a staple food in many sub-Saharan African countries, is grown by millions of resource-poor smallholder farmers. Between 2009 and 2011, maize was grown on more than 25 million hectares in sub-Saharan Africa (SSA) (Shiferaw et al. 2011), accounting for 7.5 % of global production. Average maize yield in SSA is 1.8 Mg ha^−1^, which is significantly lower than the yield in other maize-growing regions in the developing world. Recurrent drought is one of the major abiotic stresses in SSA, with approximately 22 % of mid-altitude/subtropical and 25 % of lowland tropical maize growing regions affected annually (Heisey and Edmeades 1999). Drought is expected to increase in severity due to the changing climate. Therefore, development and deployment of tropical maize germplasm with relevant agronomic and adaptive traits is key to enhance the food security and livelihoods of maize farming communities.

The ability to quickly develop germplasm with resistance to important abiotic and biotic stresses will be critical to the resilience of cropping systems in the face of climate change. Conventional breeding methods have a proven track record of improving tolerance for abiotic stresses (DTMA 2015). However, progress in breeding for drought tolerance using conventional approaches can be slow due to the polygenic nature of most stress-related traits, requiring accumulation of several quantitative trait loci (QTL) into adapted genetic backgrounds. In order to uncover and characterize genomic regions or QTLs associated with drought stress, several researchers (Veldboom and Lee 1996; Ribaut et al. 1997; Tuberosa et al. 2002; Almeida et al. 2013; Semagn et al. 2013) have reported a number of QTL for grain yield and other traits under both drought stress and well-watered conditions. In most cases, however, individual QTLs for highly polygenic traits explain only a small proportion of the phenotypic variance, and are highly dependent on genetic background and environmental conditions (Semagn et al. 2013).

Molecular marker-assisted breeding, including marker-assisted backcrossing (MABC), marker-assisted recurrent selection (MARS) and genomic selection (GS), in combination with high-throughput and precise phenotyping, doubled haploidy and year-round nurseries, can significantly accelerate the development of climate resilient maize germplasm (Prasanna et al. 2013; Xu et al. 2012). MARS is a breeding approach that aims to accumulate favorable alleles for a relatively large number of QTL in a given population using a subset of markers that are significantly associated with target traits (Bernardo 2008). GS incorporates all available marker information into a model to simultaneously predict genetic values of breeding progenies for selection, enabling accumulation of favorable alleles for major and minor QTL through multiple generations of recombination (Meuwissen et al. 2001). Recently, Beyene et al. (2015a) and Semagn et al. (2015) reported genetic gains achieved through GS and MARS in 8 and 10 tropical biparental maize populations, respectively. These studies involved genotyping 148–300 F_2:3_ (C_0_) progenies with 190–286 markers, evaluating testcrosses under WW and DS conditions and advancing selected lines using GS and MARS. In both studies, each population was represented by seed bulks containing equal amounts of seed of C_0_, C_1_, C_2_, C_3_, parents, F_1_s, and lines developed via pedigree selection. Five commercial checks were included for comparison. Beyene et al. (2015a) compared GS with pedigree selection across eight biparental tropical maize populations, and reported that the average gain per cycle from GS across eight populations was 0.086 t ha^−1^. Average grain yield of C_3_-derived hybrids was significantly higher than that of hybrids derived from C_0_. Hybrids derived from C_3_ produced 7.3 % higher grain yield than those developed through conventional pedigree breeding.

Semagn et al. (2015) reported that the average gain per cycle using MARS across 10 populations was 0.1837 t ha^−1^ under WW and 0.045 t ha^−1^ under DS conditions. Combined across DS environments, C_3_-derived hybrids produced 6.0, 8.3 and 37.8 % higher grain yields than hybrids derived from conventional pedigree breeding, parental lines and commercial checks, respectively. Across WW trials, the average grain yield of C_3_ hybrids was significantly (P < 0.05) higher than those hybrids derived C_0_, the pedigree method and the commercial checks. In both studies, the authors used a composite bulk to represent lines extracted from each selection cycle instead of using individual lines. In this study, we report the testcross performance of hybrids developed from 47 to 74 individual C_1_S_2_ lines instead of bulks derived from 10 MARS populations and evaluated under both managed drought-stress and optimum conditions. The objectives of the present study were: (1) to compare the overall gain in grain yield of all C_1_S_2_-derived hybrids with that of hybrids developed through conventional pedigree breeding method and (2) to compare yield stability of hybrids from a subset of selected populations developed through MARS and pedigree selection.

## Materials and methods

### Genetic materials

Three cycles of MARS were completed on 10 tropical biparental populations. Detailed descriptions of the methodology and the results are given elsewhere (Beyene et al. 2015b) and briefly summarized here. Testcrosses were generated by crossing the F_2:3_ families (C_0_) from each population with a single-cross tester from a complementary heterotic group and evaluated under 2–3 managed drought stresses and 3–4 well-watered conditions in Kenya, Zimbabwe and Zambia. Each C_0_ population was genotyped with 190–225 SNPs and QTL analysis was performed for each population. Three selection cycles were conducted using a subset of 55–87 SNPs that were significantly associated with grain yield and anthesis-silking interval. Selected C_0_ families were first intermated to form Cycle 1 (C_1_), followed by selfing of superior C_1_ plants for two generations to form C_1_S_1_ and C_1_S_2_. At each recurrent selection cycle, selected individuals were genotyped with the significantly associated markers to increase favorable allele frequency. In each population, the top eight families from C_0_ were also advanced using a pedigree selection scheme. The various steps followed during MARS and pedigree phenotypic selection were illustrated by Beyene et al. (2015b).

### Formation of testcrosses and phenotypic evaluation

From each population, 47–74 C_1_S_2_ lines developed through MARS, five S_5_ lines developed via phenotypic pedigree selection, and the two founder parents (P1 and P2) were crossed to a single-cross tester (CML395/CML444) at the Maize Research Station of Kenya Agriculture and Livestock Research Organization (KALRO), Kiboko, Kenya. This tester has proven to be useful in hybrid formation for subtropical and mid-altitude environments, and is also used as a parent in many commercial three-way-cross hybrids in SSA (Beyene et al. 2011, 2013). Experimental lines were used as female parents, and the single-cross tester was used as the male parent. Seeds were harvested and bulked within each female row plot for use in the testcross evaluation. Testcrosses of each population, together with five commercial checks (CZH0616, H513, WH505, DK8053, and Pioneer 3253), were evaluated in 3–5 WW and 1–3 DS locations in Kenya in 2013 and 2014. An alpha-lattice design with two replications per location was utilized for the trials. The DS trials were conducted during the dry (rain-free) season by withdrawing irrigation starting 2 weeks before flowering through harvest, whereas the WW trials were conducted during the rainy season, with supplemental irrigation applied as needed. Entries were planted in two-row plots, 5 m long, with 0.75 m spacing between rows and 0.25 m between hills. Two seeds per hill were initially planted and then thinned to one plant per hill at 3 weeks after emergence to obtain a final plant population density of 53,333 plants per hectare. Fertilizers were applied at the rate of 60 kg N and 60 kg P_2_O_5_ per ha as recommended for the area. Nitrogen was applied twice: at planting and 6 weeks after emergence. Fields were kept free of weeds by hand weeding.

### Data collection

Data on grain yield (GY), plant height (PH) and anthesis date (AD) were collected. AD was recorded as the number of days from planting to when 50 % of the plants had shed pollen. PH was measured as the distance from the base of the plant to the height of the first tassel branch. In DS trials, ears were harvested from each plot and all were shelled and weighed to determine the grain yield and percent grain moisture. In the WW experiments, ears harvested from each plot were weighed, sub-samples were shelled and grain moisture was determined on the sub-samples of grain. Grain yield was estimated assuming a shelling percentage of 80 % and adjusted to 125 g/kg moisture content.

### Statistical analysis

Analysis of variance for grain yield, anthesis date and plant height within and across DS and WW locations was performed using the PROC MIXED procedure of SAS (SAS Institute 2009) considering locations and incomplete blocks as random effects and entries as fixed effects. For each population, the analyses were performed on all entries and also on groups of entries corresponding to test-crosses involving: (i) C_1_S_2_ lines; (ii) S_5_ lines extracted through phenotypic pedigree selection; (iii) commercial checks; and (iv) founder parents used for making the original populations. Contrasts were made to compare the performance of all C_1_S_2_ and the best 10 C_1_S_2_ hybrids versus five hybrids from the conventional pedigree scheme, founder parents and five commercial check hybrids. Stability analysis was done using the linear-bilinear site regression models (SREG) (Crossa and Cornelius 1997).

## Results

The combined analysis of variance across WW and DS environments showed highly significant differences among genotypes for grain yield, plant height and anthesis date. The interactions between genotypes and environments (GE) were also significant (data not shown). For most populations, the proportion of genotype to GE variance was higher for WW than DS, indicating that GE interaction was severe under drought stress than optimum-moisture conditions (Supplementary material S1). Genotypic variance for grain yield was 23–100 % larger under WW than under DS conditions. For anthesis date, variance of genotypes was 3–74 % larger under WW than under DS conditions for eight populations, but it was 39–82 % larger under DS than under WW conditions for two other populations (Supplementary material S1). Heritability estimates for grain yield were slightly higher under WW (0.3–0.8) than under DS (0–0.5) conditions. Heritability estimates for anthesis date and plant height were considerably higher under WW than under DS conditions (Table 1).

**Table 1:** Summary of 10 biparental C1S2 populations evaluated in drought-stress (DS) and well-watered (WW) environments in Kenya

Population code	Initial cross	No. of C_1_S_2_	Number of WW locations	Number of DS locations	GY under WW (t ha^−1^)	GY under DS (t ha^−1^)	AD under WW (days)	AD under DS (days)	PH under WW (cm)	PH under DS (cm)	Heritability for GY under WW (DS)	Heritability for AD under WW (DS)	Heritability for PH under WW (DS)
1008	CML540/CML505	52	4	2	6.27	3.01	62.42	71.05	251.49	208.76	0.51 (0.25)	0.80 (0.48)	0.67 (0.64)
1015	CZL04003/CML540	47	5	2	6.36	2.14	63.85	68.97	254.39	229.72	0.75 (0.10)	0.84 (0.58)	0.88 (0.46)
1016	CML540/CZL99017	51	4	3	7.23	2.69	65.27	67.61	249.86	240.71	0.58 (0.00)	0.67 (0.73)	0.83 (0.70)
1017	CML540/CML539	47	4	3	7.91	2.93	63.49	66.75	257.68	243.58	0.68 (0.44)	0.82 (0.73)	0.80 (0.75)
1018	CML505/CZL99017	61	4	1	6.03	2.06	64.66	70.09	256.47	228.88	0.61 (0.44)	0.85 (0.34)	0.88 (0.70)
1019	CZL04008/CZL0719	68	3	2	6.24	2.85	62.18	60.15	220.16	197.16	0.72 (0.07)	0.88 (0.77)	0.83 (0.66)
1020	CML542/CZL0724	65	4	2	5.77	2.83	65.55	62.68	223.61	197.09	0.57 (0.20)	0.87 (0.64)	0.82 (0.50)
1021	CML542/CZL0719	63	5	1	6.69	2.69	65.49	68.14	222.30	196.38	0.57 (0.46)	0.87 (0.61)	0.87 (0.51)
1023	CZL0618/VL062655	74	5	1	6.79	2.16	68.22	70.72	233.90	210.64	0.62 (0.49)	0.85 (0.62)	0.87 (0.57)
1028	CZL074/VL062645	65	5	1	6.73	2.71	69.93	71.19	235.89	221.39	0.32 (0.40)	0.79 (0.22)	0.82 (0.49)

The numbers in parenthesis represent heritability estimates under DS conditions

### Grain yield under drought stress conditions

**Fig. 1:**
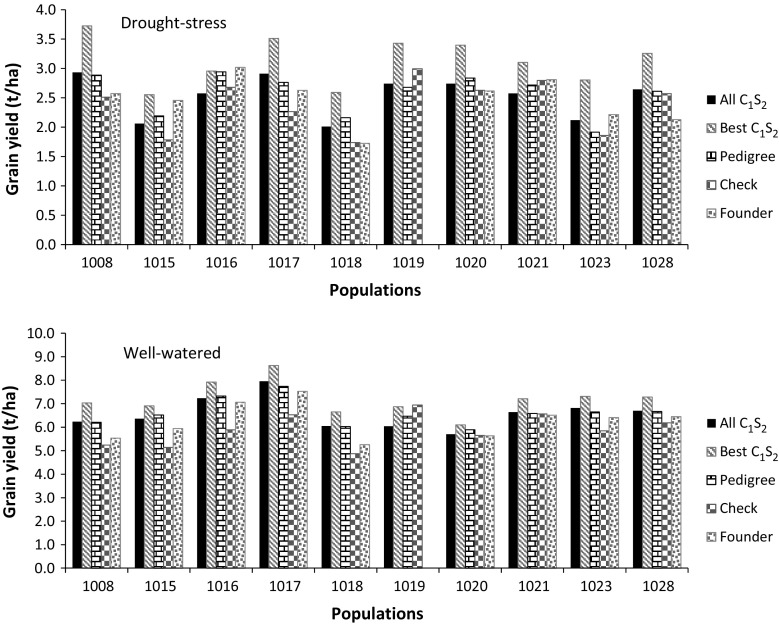
Comparison of mean grain yield of testcross of all C_1_S_2_ lines, the best 10 C_1_S_2_ lines, five lines from conventional pedigree selection, founder parents and five commercial checks evaluated in managed drought-stress and well-watered conditions in Kenya

Mean grain yields of all C_1_S_2_-derived hybrids across DS environments ranged from 2.14 to 3.01 t ha^−1^ (Table 1; Fig. 1), and the overall average was 2.61 t ha^−1^. Mean grain yield of hybrids developed from all C_1_S_2_ lines within each population was 1.7–10.8 % higher than that of hybrids derived from pedigree methods in five populations (1008, 1017, 1019, 1023 and 1028), and 3.4–12.4 % lower in the remaining five populations, but nearly all pairwise comparisons were not statistically significant (Supplementary material S2). However, each population was represented by 47–74 C_1_S_2_-derived hybrids, which is considerably higher than hybrids derived from five S_5_ lines using the pedigree method, five commercial checks and the founder parents. To make a reasonable comparison of the gains made through MARS, the best 10 C_1_S_2_ derived hybrids were compared with hybrids derived using the pedigree scheme, commercial checks and founder parents. Across DS experiments, mean grain yields of the best 10 C_1_S_2_-derived hybrids in all populations except population 1016 were significantly (*P* ≤ 0.01) higher than mean grain yields of hybrids formed from pedigree-derived lines, commercial checks, and the founder parents (Fig. 1, Supplementary material S2). Excluding population 1016, the mean grain yield of the best 10 C_1_S_2_-derived hybrids were 14.2–46.3 % (0.359–0.888 t ha^−1^), 10.3–55.1 % (0.310–1.247 t ha^−1^), and 4.0–53.0 % (0.098–1.152 t ha^−1^) higher than those of pedigree-derived hybrids, the commercial checks and the founder parents, respectively (Fig. 1, Supplementary material S2). In population 1016, the mean of the best 10 C_1_S_2_-derived hybrids produced significantly (*P* ≤ 0.05) higher grain yield (10.3 %) than the mean of the commercial checks. Combined across DS environments and all populations, the best 10 hybrids involving C_1_S_2_-derived lines produced 22.6 % (562 kg ha^−1^), 33.8 % (750 kg ha^−1^) and 27.8 % (916 kg ha^−1^) higher grain yield than hybrids formed from pedigree-derived lines, commercial checks and founder parents, respectively (Supplementary material S2).

**Fig. 2:**
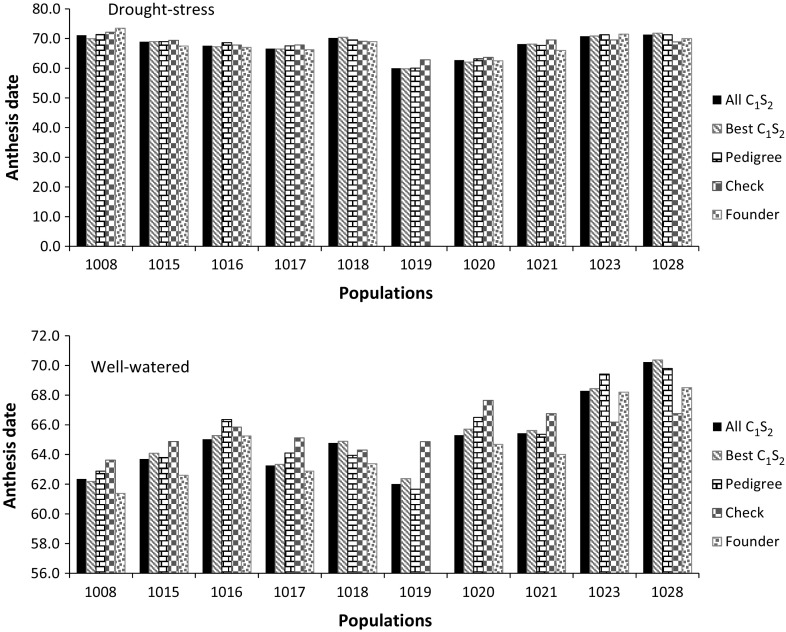
Comparison of mean anthesis date of testcrosses from all C_1_S_2_ lines, the best 10 C_1_S_2_ lines, five lines from conventional pedigree selection, founder parents and five commercial checks evaluated under managed drought-stress and well-watered conditions in Kenya

**Fig. 3:**
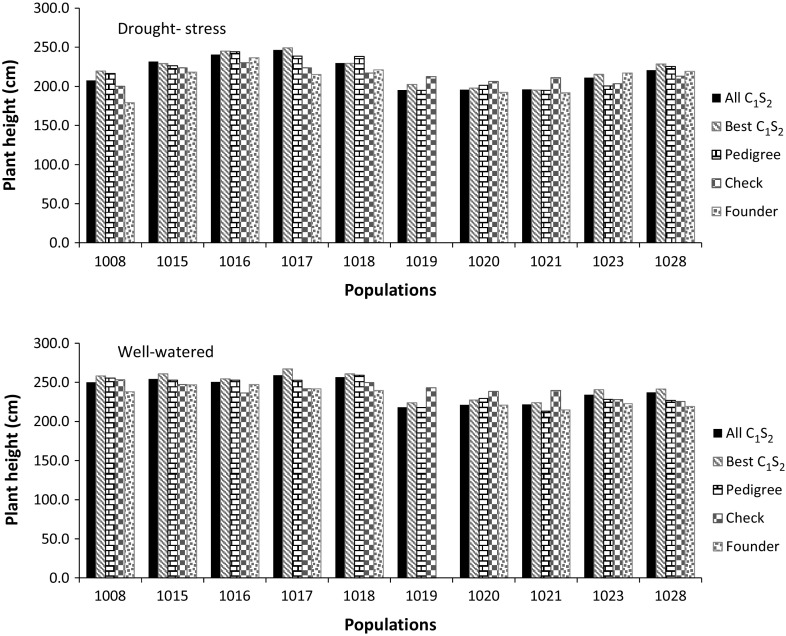
Comparison of mean plant height of testcrosses from all C_1_S_2_ lines, the best 10 C_1_S_2_ lines, five lines from conventional pedigree selection, founder parents and five commercial checks evaluated in managed drought-stress and well-watered conditions in Kenya

Although grain yield was the primary target trait, anthesis date and plant height were also analyzed to determine if grain yield gain was related to a significant change in either trait. The best 10 hybrids involving C_1_S_2_-derived lines showed a difference of 0–1.5 days to flowering with those hybrids formed from pedigree-derived lines and up to 3.5 days difference compared with both the commercial checks and the founder parents (Fig. 2, Supplementary material S2). For PH, the mean of the best 10 C_1_S_2_-derived hybrids from three populations (populations 1017, 1019, and 1023) was significantly taller than the mean of the five hybrids formed from lines derived using the pedigree method (Fig. 3, Supplementary material S2). Additionally, the mean plant heights of the best 10 hybrids involving C_1_S_2_-derived lines in nine of the ten populations were significantly higher than that of the commercial checks (Fig. 3).

### Grain yield under well-watered conditions

As shown in Table 1 and Fig. 1, mean grain yield of testcrosses evaluated in WW environments varied from 5.77 t ha^−1^ (population 1020) to 7.91 t ha^−1^ (population 1017), with an overall average of 6.60 t ha^−1^. Mean grain yields of hybrids developed from all C_1_S_2_ lines within each population showed a 0.4–2.8 % advantage over those hybrids involving pedigree-derived lines in six populations (1008, 1017, 1018, 1021, 1023 and 1028), but showed a 1.3–6.6 % reduction compared to those of the other four populations (Fig. 1, Supplementary material S2). All contrasts between the mean grain yield of the best 10 hybrids formed from C_1_S_2_-derived lines versus the mean grain yield of the five hybrids involving pedigree derived lines, the commercial checks and the founder parents were significant (*P* ≤ 0.01) (Supplementary material S2). The best 10 hybrids of C_1_S_2_-derived lines per population produced (a) 3.4–13.3 % higher grain yield than those developed using lines through the pedigree method, (b) 9.9–36.5 % higher grain yield than the commercial checks (except population 1019, which showed a 1.1 % reduction), and (c) 8.1–27.0 % higher grain yield than the founder parents. Taking into account the time invested in developing the lines and using the grain yield of the founder parents as a baseline, the top 10 C_1_S_2_-derived hybrids on average produced 214.8 kg ha^−1^ year^−1^ under WW conditions, which is approximately double the 103.9 kg ha^−1^ year^−1^ grain yield observed for hybrids developed using the pedigree method.

Mean flowering date of the best 10 hybrids of C_1_S_2_-derived lines was generally similar to those hybrids formed from pedigree-derived lines, the founder parents and the commercial checks, with a maximum difference of 1–3 days (Fig. 2, Supplementary material S2). Pairwise comparisons of mean plant height of the different groups were significant for most populations (Supplementary material S2). The best 10 hybrids of C_1_S_2_-derived lines were 6–14.5 and 11–25.3 cm taller than the hybrids formed from pedigree derived lines and the commercial checks, respectively, in six populations (Fig. 3, Supplementary material S2). Compared with the commercial checks, the best 10 hybrids involving C_1_S_2_ derived lines were 11.0–19.2 cm shorter in three populations (1019, 1020, and 1021).

### Grain yield stability

**Fig. 4:**
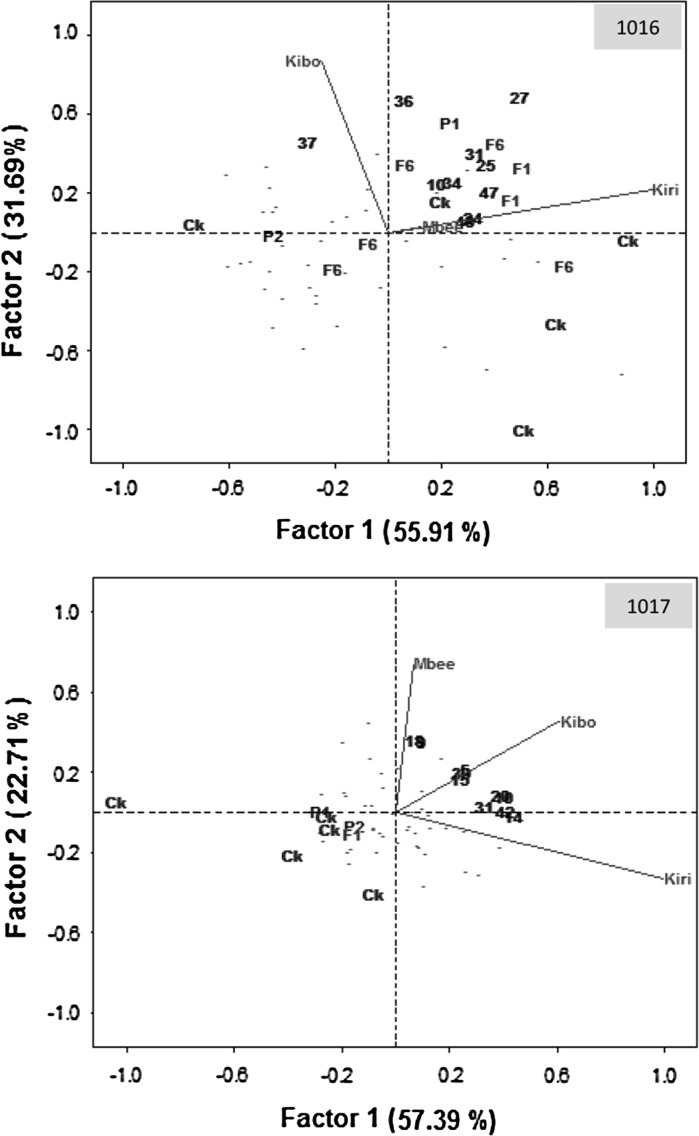
Biplot of the site regression model (SREG) for two biparental populations evaluated in three managed drought-stressed sites (Kiboko, Kiri and Mbee) in Kenya. Each population is represented by the best 10 hybrids derived from C_1_S_2_ (*black numbers*) (other entries from the C_1_S_2_ are represented by a *black dot*), commercial checks (abbreviated as Ck in *green color*), the 5 hybrids derived from the pedigree method (F6 in *red*) and founder parents P1 and P2 (*light blue*). (Color figure online)

To compare grain yield stability of the best 10 hybrids of C_1_S_2_-derived lines with that of hybrids formed from pedigree derived lines, commercial checks and founders, we selected two populations (1016 and 1017) that were evaluated in three DS locations, and four populations (1015, 1021, 1023 and 1028) that were evaluated in five WW locations. Figure 4 summarizes biplots of the grain yield of the two populations evaluated under DS conditions. The first two axes from the GGE biplot for populations 1016 and 107 explained 87.6 and 80.1 %, respectively, of the genotypic main effect. The two-dimensional biplot showed that almost all of the best 10 hybrids of C_1_S_2_-derived lines had positive PC1 scores, suggesting they had above average performance. In population 1016, four hybrids derived from C_1_S_2_ (entries 10, 34, 25,31 and 47) were high yielding and stable, with high PC1 scores and near-zero PC2 scores.

**Fig. 5:**
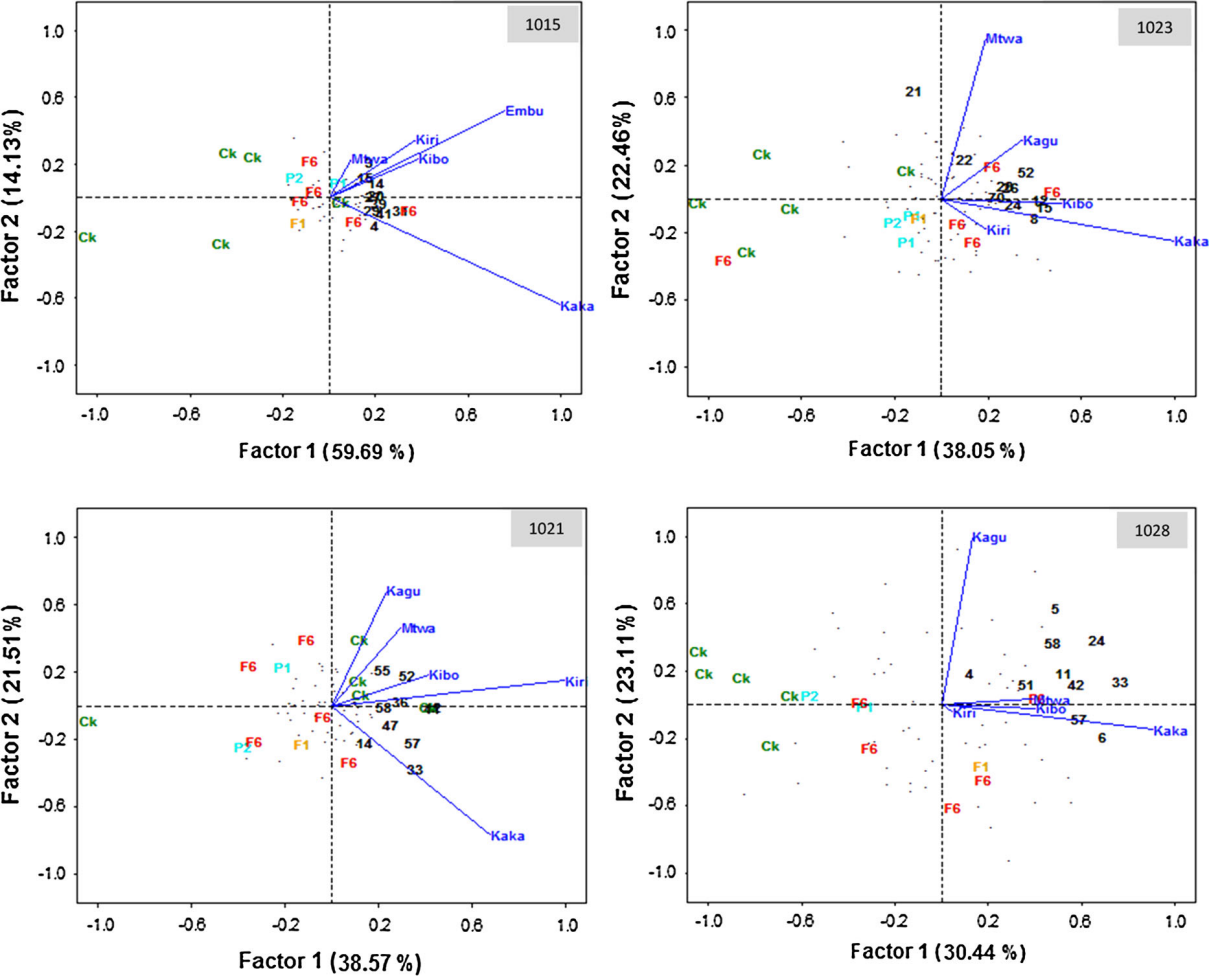
Biplot of the site regression model (SREG) for four biparental populations evaluated in five well-watered sites in Kenya. Each population is represented by the best 10 hybrids derived from C_1_S_2_ (*black numbers*) other C_1_S_2_ entries are represented by a *black dot*, commercial checks (abbreviated as Ck and given in *green color*), the 5 hybrids derived from the pedigree method (F6 in *red*) and founder parents P1 and P2 (*light blue*). (Color figure online)

The superior grain yield of most of the best 10 hybrids of C_1_S_2_-derived lines over the five hybrids involving pedigree-derived lines is depicted in Figs. 4 and 5. The majority of the top hybrids derived from C_1_S_2_ were consistently located towards the upper right quadrant of the biplots, indicating that those entries had both a positive interaction with those environments and higher mean grain yield than entries located on the left-hand side of the biplots (opposite to the direction of the sites). The GGE biplot for populations 1015, 1021, 1023 and 1028 evaluated in five WW environments explained 53.5–73.8 % of the genotypic main effect (Fig. 5). The two-dimensional biplot showed that almost all of the best 10 hybrids of C_1_S_2_-derived lines had positive PC1 scores, suggesting above average performance, while most pedigree derived hybrids and the commercial checks had negative PC1 scores, indicating below average performance. Embu, Kakamega and Kaguru had longer vectors than the other locations, suggesting that they were the best locations for discriminating hybrids. In population 1023, most hybrids (entries 11, 42, 23, 6 and 57) had high and stable yields, as they have high positive PC1 scores and near zero PC2 scores. In some populations, the best 10 hybrids of C_1_S_2_-derived lines that had high grain yield under DS were also found to be among the best 10 under WW conditions (Table 2). For example, entries 27, 36 and 37 from population 1016 were among the best 10 hybrids of C_1_S_2_-derived lines in both DS and WW locations (Fig. 5; Table 2).

**Table 2:** Entry code of the best 10 C_1_S_2_ derived hybrids and their grain yield (GY) (t ha^−1^) in water-stress and well-watered locations for each of the ten biparental populations

Drought-stress		Well-watered		Drought-stress		Well-watered	
Entry	GY	Entry	GY	Entry	GY	Entry	GY
Population 1008				Population 1015			
43	4.008	***14***	7.997	47	3.196	20	7.089
46	3.891	48	7.909	2	3.119	31	7.059
6	3.801	41	7.713	10	2.614	3	7.037
17	3.722	42	7.669	19	2.490	14	6.932
***14***	3.622	5	7.608	22	2.415	27	6.867
49	3.556	35	7.576	38	2.375	29	6.840
13	3.551	18	7.550	42	2.371	15	6.825
***25***	3.545	1	7.550	25	2.365	4	6.797
27	3.541	7	7.541	44	2.359	39	6.795
21	3.502	***25***	7.468	46	2.353	41	6.767
Population 1016				Population 1017			
***36***	3.068	***37***	8.672	42	4.352	25	8.905
47	3.024	31	8.473	10	4.300	24	8.672
48	3.021	30	8.239	***14***	4.268	***27***	8.653
***27***	2.994	***27***	8.167	20	4.246	11	8.581
24	2.952	***36***	8.129	17	4.069	40	8.560
10	2.914	35	8.057	16	4.060	28	8.555
***37***	2.913	26	8.008	31	4.015	34	8.492
25	2.903	44	7.963	19	4.014	***14***	8.487
34	2.877	34	7.937	***27***	3.943	26	8.464
31	2.863	32	7.923	5	3.935	4	8.463
Population 1018				Population 1019			
***8***	2.831	***8***	7.038	***52***	2.890	2	7.370
52	2.651	***60***	6.939	56	2.881	1	7.121
***60***	2.647	44	6.858	41	2.822	12	7.069
4	2.639	42	6.616	42	2.790	3	7.046
36	2.574	10	6.603	1	2.786	50	6.821
35	2.542	38	6.586	***29***	2.712	23	6.748
46	2.462	53	6.565	9	2.640	***29***	6.643
14	2.455	7	6.543	55	2.624	51	6.634
41	2.427	22	6.542	11	2.605	***52***	6.617
32	2.420	30	6.488	22	2.527	10	6.604
Population 1020				Population 1021			
42	3.181	5	6.374	54	3.231	44	7.415
63	2.960	***25***	6.368	63	3.223	55	7.412
***40***	2.914	12	6.257	57	3.218	33	7.368
36	2.726	29	6.180	14	3.153	12	7.309
62	2.676	62	6.169	51	3.086	57	7.189
45	2.642	28	6.164	27	3.066	36	7.178
***25***	2.620	***40***	6.149	7	3.053	14	7.149
53	2.578	57	6.132	39	3.019	52	7.137
50	2.511	63	6.128	40	2.997	58	7.121
19	2.507	4	6.105	6	2.993	47	7.115
Population 1023				Population 1028			
51	3.944	12	7.583	19	3.561	33	7.602
72	2.919	***20***	7.574	16	3.489	24	7.511
***20***	2.849	52	7.388	59	3.363	57	7.363
41	2.722	70	7.300	60	3.362	51	7.326
***26***	2.713	60	7.288	7	3.183	5	7.323
35	2.682	***26***	7.218	18	3.180	4	7.265
17	2.678	22	7.207	54	3.103	42	7.242
23	2.610	11	7.154	12	3.085	6	7.208
14	2.609	46	7.152	11	3.062	11	7.199
25	2.605	24	7.142	61	3.059	58	7.176

Bolditalic indicates the entry number of hybrids that performed well both under WW and DS conditions

## Discussion

Conventional pedigree breeding has been used successfully to develop improved maize germplasm with abiotic and biotic stress resilience. Since 2007, CIMMYT and partners have used conventional breeding methods to develop and release over 200 drought tolerant hybrids and open-pollinated maize varieties in SSA under the framework of the Drought Tolerant Maize for Africa project (DTMA 2015). However, developing improved varieties using conventional breeding methods takes long, since many economically important traits require simultaneous accumulation of favorable alleles from several genomic regions. The use of molecular markers within breeding pipelines is widely, and successfully, employed by large private sector companies (Johnson 2004; Eathington et al. 2007). Use of molecular markers for tropical maize improvement in the developing world is however, constrained by several bottlenecks (Xu et al. 2012; Mba et al. 2012).

To facilitate the development and use of improved tropical maize germplasm, CIMMYT in collaboration with public and private partners conducted the largest public MARS and GS projects as part of both the Water Efficient Maize for Africa (WEMA) and DTMA projects. Based on the genetic gain data collected by these projects across multiple populations, each represented by a composite bulk of lines, we recently demonstrated the superiority of MARS for increasing grain yield under DS and WW conditions across diverse tropical maize populations without significantly affecting plant height and maturity of most populations (Beyene et al. 2015a; Semagn et al. 2015). When genetic gains for individual populations were considered, results indicated that different populations showed deferent responses to MARS, with the majority of the populations producing higher grain yields than those developed using conventional breeding methods. A smaller number of populations showed either similar or no gain in grain yield under both WW and DS conditions as compared with those developed through pedigree selection. A possible factor contributing to the lack of gain in grain yield from those populations may be the representation of every population by a composite bulk prior to testcross formation, which was implemented primarily to minimize the number of entries to be evaluated.

In the present study, hybrids developed by crossing 47–74 C_1_S_2_ lines from each of the 10 MARS populations with a single cross tester were evaluated. Combined across all DS locations and populations, the best 10 C_1_S_2_-derived hybrids produced 562, 750 and 916 kg ha^−1^ higher grain yield than pedigree-derived hybrids, commercial checks and founder parents, respectively. In WW locations, the best 10 C_1_S_2_-derived hybrids produced 583, 1305 and 1557 kg ha^−1^ more grain yield than pedigree-derived hybrids, commercial checks and founder parents, respectively.

As previously described by Beyene et al. (2015b) 3.5 years were required to develop S_5_ lines through pedigree selection and 4 years to develop C_1_S_2_ lines through MARS. Considering the number of years spent in developing the lines used in the study and taking the grain yield of the founder parents as the baseline data, the top 10 hybrids of C_1_S_2_-derived lines on average yielded 229 and 389.3 kg ha^−1^ year^−1^ in DS and WW conditions, respectively, which is higher than the yield of the hybrids formed from pedigree-derived lines (27.2 kg ha^−1^ year^−1^ under DS and 103.9 kg ha^−1^ year^−1^ under WW). The overall gains from the best 10 hybrids of C_1_S_2_-derived lines in the present study were higher than previous results reported by Beyene et al. (2015b) using composite bulk sampling. Although composite bulking of C_1_S_2_ lines prior to hybrid formation and their evaluation under multiple environments provided an overall idea of the genetic gain obtained through MARS over pedigree selection, results of the present study clearly demonstrate the value of evaluating every C_1_S_2_-derived hybrid to identify the best lines for further inbreeding and hybrid development. As shown in Supplementary material S2, the top 10 hybrids of C_1_S_2_-derived lines from each of the 10 biparental populations produced higher mean grain yields than hybrids developed using pedigree derived lines This result is similar to the findings of Eathington et al. (2007), who compared MARS and conventional selection in 248 North American and European maize breeding populations, and reported higher performance and yield index gains for MARS that was more than doubled compared to phenotypic selection. They also found that the MARS-derived lines were higher performing compared to conventionally selected lines.

A recent review of genetic gain studies from conventional pedigree selection conducted both in temperate and tropical maize germplasm reported highly variable results (Edmeades 2013). In SSA, preliminary estimates of yield gains from conventional selection revealed 39–80 kg ha^−1^ year^−1^ under optimal conditions, but only 18 kg ha^−1^ year^−1^ under drought stress (Edmeades 2013). A recent study using 67 hybrids developed at CIMMYT and released between 2000 and 2011 showed genetic gains of 32 and 109 kg ha^−1^ year^−1^ for grain yield under managed drought and well-watered conditions, respectively (Masuka et al. 2015, submitted). Therefore, the overall average gain obtained under DS (229 kg ha^−1^ year^−1^) and WW (389.3 kg ha^−1^ year^−1^) in the current study was three to seven times higher than that reported from conventional phenotypic selection in SSA. Genetic gains obtained through pedigree breeding in the current study (27.2 and 103.9 kg ha^−1^ year^−1^ under DS and WW conditions, respectively) were similar to estimates reported in the literature in SSA (Edmeades 2013).

Edmeades et al. (2004) reported that the phenotypic correlation between elite hybrid yields under stress versus under well-watered conditions declined as stress intensified, reaching 0.35 (*r*^*2*^ = 0.12) when yield reductions reached 50 %. They suggested that stress adaptive mechanisms were not exposed until yields had been reduced by 30–50 % under stress. In the current study, the average grain yield were 6.60 t ha^−1^ under WW condition and 2.61 t ha^−1^ under DS condition, which represented a 61 % yield reduction, approaching the 70 % yield reduction typically targeted by CIMMYT in managed drought stress experiments in SSA (Bänziger et al. 2000). Accordingly, the best 10 C_1_C_2_ lines identified in these studies from each population may have adaptive traits for drought tolerance, which could be utilized as sources of drought tolerance in maize breeding.

Relative differences in genetic gains observed between MARS and pedigree selection were much higher under DS conditions than under WW conditions), suggesting that MARS could accelerate the pace of improvement, for complex traits such as drought tolerance. These results agree with previous reports (Eathington et al. 2007; Xu and Crouch 2008; Beyene et al. 2015b), indicating that MARS can be more efficient and effective than phenotypic selection, and could improve genetic gains for complex traits like drought tolerance in tropical maize breeding programs.

Since drought incidence and severity vary considerably among years and within fields, it is important to develop hybrids that are able to withstand drought stress throughout the growing season, but also have no yield penalty under optimum conditions. Hybrids performing well under both DS and WW conditions were identified in this study (Table 2). For example, entries 14 and 25 in population 1008 and entries 27, 36, and 37 in population 1016 are among the top performing hybrids under both DS and WW conditions. Therefore, the parents of these hybrids need to be fixed through generation of inbreeding to develop hybrids that will perform better both under drought stress and well water conditions.

## Electronic supplementary material

Below is the link to the electronic supplementary material.

Supplementary material 1 (DOCX 46 KB)
