# Supplementary material for: Performance and grain yield stability of maize populations developed using marker-assisted recurrent selection and pedigree selection procedures
Source: Euphytica. 2015 Nov 9;208:285–97. doi: 10.1007/s10681-015-1590-1 (PMC4913958; doi:10.1007/s10681-015-1590-1)
Supplement: Supplementary file 1 — Supplementary material 1 (DOCX 46 KB) [file 10681_2015_1590_MOESM1_ESM.docx]

**Supplementary materials**

**Supplementary material S1**. Location, entry, location × entry, and residual variance components for three traits (grain yield, anthesis days and plant height) combined across water stress (DS) and well-watered (WW) locations in each of the 10 biparental populations.

| Variance component | Grain yield  (t/ha) | Anthesis day  (days) | Plant height  (cm) | Grain yield  (t/ha) | Anthesis day  (days) | Plant height  (cm) |
| --- | --- | --- | --- | --- | --- | --- |
|  | DS | | | WW | | |
|  | -------------------------------Population 1008----------------------------------- | | | | | |
| Location | 0.491 | 15.91 | 729.00 | 6.770 | 70.55 | 131.32 |
| Entry | 0.057 | 1.24 | 70.08 | 0.144 | 0.68 | 49.03 |
| Loc × Entry | 0.056 | 0.00 | 0.00 | 0.182 | 0.21 | 13.86 |
| Residual | 0.573 | 5.41 | 160.70 | 0.752 | 0.97 | 116.25 |
|  | --------------------------------Population 1015---------------------------------- | | | | | |
| Location | 0.086 | 0.00 | 0.00 | 3.591 | 65.15 | 5.52 |
| Entry | 0.011 | 0.28 | 31.52 | 0.262 | 0.80 | 82.06 |
| Loc × Entry | 0.056 | 0.02 | 4.55 | 0.185 | 0.24 | 3.92 |
| Residual | 0.261 | 0.36 | 66.05 | 0.526 | 0.99 | 78.47 |
|  | ---------------------------------Population 1016--------------------------------- | | | | | |
| Location | 1.287 | 9.25 | 0.00 | 2.593 | 118.17 | 591.74 |
| Entry | 0.000 | 0.68 | 69.84 | 0.207 | 0.49 | 68.01 |
| Loc × Entry | 0.067 | 0.17 | 14.69 | 0.243 | 0.28 | 0.00 |
| Residual | 0.206 | 0.65 | 91.92 | 0.710 | 1.38 | 81.28 |
|  | ---------------------------------Population 1017--------------------------------- | | | | | |
| Location | 1.101 | 4.70 | 184.98 | 2.875 | 140.03 | 80.42 |
| Entry | 0.066 | 0.86 | 86.50 | 0.317 | 0.89 | 77.21 |
| Loc × Entry | 0.080 | 0.21 | 4.06 | 0.212 | 0.26 | 13.03 |
| Residual | 0.350 | 0.83 | 108.13 | 0.766 | 1.03 | 90.06 |
|  | ------------------------------Population 1018------------------------------------ | | | | | |
| Location | 0.000 | 0.00 | 0.00 | 6.144 | 74.74 | 1.79 |
| Entry | 0.042 | 0.18 | 85.41 | 0.170 | 0.70 | 107.91 |
| Loc × Entry | 0.002 | 0.00 | 1.10 | 0.223 | 0.12 | 2.40 |
| Residual | 0.105 | 0.70 | 71.05 | 0.431 | 0.73 | 86.05 |
|  | ------------------------------Population 1019------------------------------------ | | | | | |
| Location | 1.076 | 59.05 | 0.00 | 1.339 | 63.09 | 49.46 |
| Entry | 0.007 | 1.17 | 65.49 | 0.215 | 1.58 | 103.92 |
| Loc × Entry | 0.033 | 0.10 | 6.05 | 0.050 | 0.16 | 19.23 |
| Residual | 0.312 | 1.23 | 55.19 | 0.408 | 0.98 | 85.50 |
|  | ----------------------------------------1020--------------------------------------- | | | | | |
| Location | 1.012 | 63.83 | 0.00 | 3.087 | 59.40 | 90.90 |
| Entry | 0.025 | 0.69 | 36.23 | 0.112 | 1.05 | 52.07 |
| Loc × Entry | 0.000 | 0.01 | 2.62 | 0.093 | 0.20 | 2.52 |
| Residual | 0.402 | 1.53 | 66.09 | 0.505 | 0.85 | 85.95 |
|  | ---------------------------------Population 1021-------------------------------- | | | | | |
| Location | 0.000 | 0.00 | 0.00 | 1.468 | 154.56 | 357.10 |
| Entry | 0.044 | 0.67 | 41.03 | 0.096 | 0.85 | 66.91 |
| Loc × Entry | 0.000 | 0.06 | 15.93 | 0.111 | 0.18 | 0.00 |
| Residual | 0.101 | 0.73 | 47.71 | 0.495 | 0.93 | 80.75 |
|  | ------------------------------------Population 1023------------------------------ | | | | | |
| Location | 0.000 | 0.00 | 0.00 | 5.578 | 78.65 | 211.32 |
| Entry | 0.070 | 0.51 | 38.35 | 0.123 | 0.79 | 66.73 |
| Loc × Entry | 0.001 | 0.01 | 0.04 | 0.064 | 0.07 | 0.00 |
| Residual | 0.146 | 0.59 | 56.91 | 0.634 | 1.30 | 79.32 |
|  | ---------------------------------Population 1028--------------------------------- | | | | | |
| Location | 0.000 | 0.00 | 0.00 | 1.865 | 99.81 | 359.96 |
| Entry | 0.032 | 1.06 | 29.53 | 0.042 | 1.34 | 57.65 |
| Loc × Entry | 0.000 | 0.01 | 0.08 | 0.199 | 0.46 | 0.34 |
| Residual | 0.097 | 7.29 | 61.44 | 0.548 | 2.59 | 102.33 |

| **Supplementary material S2**. Probability-*F* and means of testcrosses from all C_1_S_2_ lines_,_ the best 10 C_1_S_2_ lines, five S_5_ lines from pedigree selection, founder parents, and five commercial checks for 10 populations. G grain yield (GY, t ha^-1^), anthesis days (AD, days) and plant height (PH, cm) were evaluated in drought-stress and well-watered sites. | | | | | | | | | | | | | | | | | | | | | | | | | | | | | | | | | | | |  |  |  |  |
| --- | --- | --- | --- | --- | --- | --- | --- | --- | --- | --- | --- | --- | --- | --- | --- | --- | --- | --- | --- | --- | --- | --- | --- | --- | --- | --- | --- | --- | --- | --- | --- | --- | --- | --- | --- | --- | --- | --- | --- |
|  | | | GY | | AD | | | | PH | | | | |  | | | | | | GY | | | | | AD | | | | | PH | | | | | |  |  |  |  |
|  | | | Drought -stress-------- | | | | | | | | | | |  | | | | | | -------- Well-watered ----- | | | | | | | | | | | | | | | |  |  |  |  |
|  | | | ---------------------Population 1008 --------------------- | | | | | | | | | | | | | | | | | | | | | | | | | | | | | | | | |  |  |  |  |
|  | | | --------------------------- *Probability-P* ----------------------------- | | | | | | | | | | | | | | | | | | | | | | | | | | | | | | | | |  |  |  |  |
| All C_1_S_2_ vs Pedigree | | | 0.22 | | 0.43 | | | | 0.19 | | | | |  | | | | | | 0.09 | | | | | 0.01 | | | | | 0.40 | | | | | |  |  |  |  |
| All C_1_S_2_ vs Checks | | | 0.05 | | 0.03 | | | | 0.06 | | | | |  | | | | | | <.0001 | | | | | <.0001 | | | | | 0.96 | | | | | |  |  |  |  |
| All C_1_S_2_ vs Parents | | | 0.19 | | 0.04 | | | | 0.00 | | | | |  | | | | | | 0.08 | | | | | 0.00 | | | | | 0.21 | | | | | |  |  |  |  |
| Best C_1_S_2_ vs Pedigree | | | 0.00 | | 0.03 | | | | 0.28 | | | | |  | | | | | | <.0001 | | | | | 0.01 | | | | | 0.40 | | | | | |  |  |  |  |
| Best C_1_S_2_ vs Checks | | | <.0001 | | 0.00 | | | | <.0001 | | | | |  | | | | | | <.0001 | | | | | <.0001 | | | | | 0.11 | | | | | |  |  |  |  |
| Best C_1_S_2_ vs Parents | | | 0.01 | | 0.01 | | | | <.0001 | | | | |  | | | | | | 0.00 | | | | | 0.01 | | | | | 0.04 | | | | | |  |  |  |  |
|  | | | ------------------------------- Mean -------------------------------------- | | | | | | | | | | | | | | | | | | | | | | | | | | | | | | | | |  |  |  |  |
| All C_1_S_2_ | | | 2.9 | | 71.1 | | | | 207.5 | | | | |  | | | | | | 6.2 | | | | | 62.4 | | | | | 250.0 | | | | | |  |  |  |  |
| Best C_1_S_2_ | | | 3.7 | | 69.9 | | | | 219.5 | | | | |  | | | | | | 7.0 | | | | | 62.2 | | | | | 258.1 | | | | | |  |  |  |  |
| Pedigree | | | 2.9 | | 71.4 | | | | 216.4 | | | | |  | | | | | | 6.2 | | | | | 62.9 | | | | | 255.5 | | | | | |  |  |  |  |
| Check | | | 2.5 | | 72.2 | | | | 200.2 | | | | |  | | | | | | 5.2 | | | | | 63.6 | | | | | 253.2 | | | | | |  |  |  |  |
| Parents | | | 2.6 | | 73.5 | | | | 179.1 | | | | |  | | | | | | 5.5 | | | | | 61.4 | | | | | 238.1 | | | | | |  |  |  |  |
|  | | |  | | | | | | | | | | | | | | | | | | | | | | | | | | | | | | | | |  |  |  |  |
|  | | | ----------------------Population 1015--------------------- | | | | | | | | | | | | | | | | | | | | | | | | | | | | | | | | |  |  |  |  |
|  | | | ------------------------- *Probability-P* ---------------------------- | | | | | | | | | | | | | | | | | | | | | | | | | | | | | | | | |  |  |  |  |
| All C_1_S_2_ vs Pedigree | | | 0.81 | | 0.76 | | | | 0.14 | | | | |  | | | | | | 0.75 | | | | | 0.98 | | | | | 0.01 | | | | | |  |  |  |  |
| All C_1_S_2_ vs Checks | | | 0.00 | | 0.08 | | | | 0.01 | | | | |  | | | | | | <.0001 | | | | | <.0001 | | | | | 0.00 | | | | | |  |  |  |  |
| All C_1_S_2_ vs Parents | | | 0.47 | | 0.00 | | | | 0.01 | | | | |  | | | | | | 0.06 | | | | | 0.00 | | | | | 0.05 | | | | | |  |  |  |  |
| Best C_1_S_2_ vs Pedigree | | | 0.01 | | 0.88 | | | | 0.37 | | | | |  | | | | | | 0.01 | | | | | 0.12 | | | | | <.0001 | | | | | |  |  |  |  |
| Best C_1_S_2_ vs Checks | | | <.0001 | | 0.16 | | | | 0.08 | | | | |  | | | | | | <.0001 | | | | | <.0001 | | | | | <.0001 | | | | | |  |  |  |  |
| Best C_1_S_2_ vs Parents | | | 0.44 | | 0.00 | | | | 0.03 | | | | |  | | | | | | 0.00 | | | | | <.0001 | | | | | 0.00 | | | | | |  |  |  |  |
|  | | | ---------------------------------- Mean ---------------------------------- | | | | | | | | | | | | | | | | | | | | | | | | | | | | | | | | |  |  |  |  |
| All C_1_S_2_ | | | 2.1 | | 69.0 | | | | 231.6 | | | | |  | | | | | | 6.4 | | | | | 63.7 | | | | | 254.3 | | | | | |  |  |  |  |
| Best C_1_S_2_ | | | 2.6 | | 69.0 | | | | 229.3 | | | | |  | | | | | | 6.9 | | | | | 64.1 | | | | | 260.9 | | | | | |  |  |  |  |
| Pedigree | | | 2.2 | | 69.0 | | | | 226.4 | | | | |  | | | | | | 6.5 | | | | | 63.8 | | | | | 252.8 | | | | | |  |  |  |  |
| Check | | | 1.8 | | 69.4 | | | | 223.6 | | | | |  | | | | | | 5.1 | | | | | 64.9 | | | | | 247.1 | | | | | |  |  |  |  |
| Parents | | | 2.5 | | 67.5 | | | | 218.3 | | | | |  | | | | | | 5.9 | | | | | 62.6 | | | | | 246.9 | | | | | |  |  |  |  |
|  | --------- Drought -stress-------- | | | | | | | | | | | | | | |  | | | | | | -------- Well-watered ----- | | | | | | | | | | | | | | | |  |  |
|  | ---------------------Population 1016 ------------------------- | | | | | | | | | | | | | | | | | | | | | | | | | | | | | | | | | | | | |  |  |
|  | ------------------------------- *Probability-P* -------------------------------- | | | | | | | | | | | | | | | | | | | | | | | | | | | | | | | | | | | | |  |  |
| All C_1_S_2_ vs Pedigree | | | | 0.00 | | | | <.0001 | | | | | 0.21 | | | | |  | | | | | | 0.96 | | | | | <.0001 | | | | | | 0.72 | | | | |
| All C_1_S_2_ vs Checks | | | | 1.00 | | 0.09 | | | | | | <.0001 | | | |  | | | | | | <.0001 | | | | | | 0.00 | | | | | <.0001 | | | | |  |  |
| All C_1_S_2_ vs Parents | | | | 0.09 | | 0.10 | | | | | | 0.48 | | | |  | | | | | | 0.19 | | | | | | 0.75 | | | | | 0.05 | | | | |  |  |
| Best C_1_S_2_ vs Pedigree | | | | 0.90 | | <.0001 | | | | | | 0.96 | | | |  | | | | | | 0.00 | | | | | | <.0001 | | | | | 0.23 | | | | |  |  |
| Best C_1_S_2_ vs Checks | | | | 0.02 | | 0.02 | | | | | | <.0001 | | | |  | | | | | | <.0001 | | | | | | 0.07 | | | | | <.0001 | | | | |  |  |
| Best C_1_S_2_ vs Parents | | | | 0.92 | | 0.38 | | | | | | 0.15 | | | |  | | | | | | 0.00 | | | | | | 0.74 | | | | | 0.00 | | | | |  |  |
|  | | | | ------------------------------------- Mean ------------------------------------- | | | | | | | | | | | | | | | | | | | | | | | | | | | | | | | | | |  |  |
| All C_1_S_2_ | | | | 2.6 | | 67.6 | | | | | | 240.6 | | | |  | | | | | | 7.2 | | | | | | 65.0 | | | | | 250.6 | | | | |  |  |
| Best C_1_S_2_ | | | | 3.0 | | 67.3 | | | | | | 245.1 | | | |  | | | | | | 7.9 | | | | | | 65.3 | | | | | 254.5 | | | | |  |  |
| Pedigree | | | | 2.9 | | 68.6 | | | | | | 244.2 | | | |  | | | | | | 7.3 | | | | | | 66.4 | | | | | 252.8 | | | | |  |  |
| Check | | | | 2.7 | | 67.8 | | | | | | 230.2 | | | |  | | | | | | 5.9 | | | | | | 65.9 | | | | | 236.4 | | | | |  |  |
| Parents | | | | 3.0 | | 67.0 | | | | | | 236.6 | | | |  | | | | | | 7.1 | | | | | | 65.3 | | | | | 247.2 | | | | |  |  |
|  | | | |  | | | | | | | | | | | | | | | | | | | | | | | | | | | | | | | | | |  |  |
|  | | | | -----------------------Population 1017 ------------------------ | | | | | | | | | | | | | | | | | | | | | | | | | | | | | | | | | |  |  |
|  | | | | -------------------------------- *Probability-P* --------------------------- | | | | | | | | | | | | | | | | | | | | | | | | | | | | | | | | | |  |  |
| All C_1_S_2_ vs Pedigree | | | | 0.02 | | <.0001 | | | | | | 0.01 | | | |  | | | | | | 0.01 | | | | | | 0.00 | | | | | 0.00 | | | | |  |  |
| All C_1_S_2_ vs Checks | | | | <.0001 | | <.0001 | | | | | | <.0001 | | | |  | | | | | | <.0001 | | | | | | <.0001 | | | | | <.0001 | | | | |  |  |
| All C_1_S_2_ vs Parents | | | | 0.05 | | 0.34 | | | | | | 0.00 | | | |  | | | | | | 0.08 | | | | | | 0.39 | | | | | 0.00 | | | | |  |  |
| Best C_1_S_2_ vs Pedigree | | | | <.0001 | | <.0001 | | | | | | 0.01 | | | |  | | | | | | <.0001 | | | | | | 0.01 | | | | | <.0001 | | | | |  |  |
| Best C_1_S_2_ vs Checks | | | | <.0001 | | <.0001 | | | | | | <.0001 | | | |  | | | | | | <.0001 | | | | | | <.0001 | | | | | <.0001 | | | | |  |  |
| Best C_1_S_2_ vs Parents | | | | 0.00 | | 0.46 | | | | | | 0.00 | | | |  | | | | | | 0.01 | | | | | | 0.27 | | | | | <.0001 | | | | |  |  |
|  | | | | --------------------------------------- Mean ----------------------------------- | | | | | | | | | | | | | | | | | | | | | | | | | | | | | | | | | |  |  |
| All C_1_S_2_ | | | | 2.9 | | 66.6 | | | | | | 246.5 | | | |  | | | | | | 8.0 | | | | | | 63.3 | | | | | 259.0 | | | | |  |  |
| Best C_1_S_2_ | | | | 3.5 | | 66.5 | | | | | | 249.3 | | | |  | | | | | | 8.6 | | | | | | 63.3 | | | | | 267.2 | | | | |  |  |
| Pedigree | | | | 2.8 | | 67.6 | | | | | | 238.7 | | | |  | | | | | | 7.7 | | | | | | 64.1 | | | | | 252.8 | | | | |  |  |
| Check | | | | 2.3 | | 67.9 | | | | | | 223.6 | | | |  | | | | | | 6.5 | | | | | | 65.1 | | | | | 241.9 | | | | |  |  |
| Parents | | | | 2.6 | | 66.3 | | | | | | 215.1 | | | |  | | | | | | 7.5 | | | | | | 62.9 | | | | | 241.8 | | | | |  |  |
|  | | --------- Drought-stress------- | | | | | | | | | | | | |  | | | | | | | | ---------Well-watered----- | | | | | | | | | | | | | |  |  |  |
|  | | -----------------------Population 1018 ---------------------- | | | | | | | | | | | | | | | | | | | | | | | | | | | | | | | | | | |  |  |  |
|  | | ------------------------------ *Probability-P* ----------------------------- | | | | | | | | | | | | | | | | | | | | | | | | | | | | | | | | | | |  |  |  |
| All C_1_S_2_ vs Pedigree | | 0.72 | | | | | 0.02 | | | 0.02 | | | | |  | | 0.12 | | | | | | | | | <.0001 | | | | | 0.42 | | | | | |  |  |  |
| All C_1_S_2_ vs Checks | | 0.00 | | | | | 0.00 | | | 0.00 | | | | |  | | <.0001 | | | | | | | | | 0.00 | | | | | 0.00 | | | | | |  |  |  |
| All C_1_S_2_ vs Parents | | 0.22 | | | | | 0.04 | | | 0.27 | | | | |  | | 0.00 | | | | | | | | | 0.00 | | | | | 0.00 | | | | | |  |  |  |
| Best C_1_S_2_ vs Pedigree | | 0.02 | | | | | 0.01 | | | 0.07 | | | | |  | | <.0001 | | | | | | | | | <.0001 | | | | | 0.56 | | | | | |  |  |  |
| Best C_1_S_2_ vs Checks | | <.0001 | | | | | <.0001 | | | 0.00 | | | | |  | | <.0001 | | | | | | | | | 0.00 | | | | | <.0001 | | | | | |  |  |  |
| Best C_1_S_2_ vs Parents | | 0.01 | | | | | 0.02 | | | 0.23 | | | | |  | | <.0001 | | | | | | | | | <.0001 | | | | | <.0001 | | | | | |  |  |  |
|  | | ------------------------------------ Mean ----------------------------------- | | | | | | | | | | | | | | | | | | | | | | | | | | | | | | | | | | |  |  |  |
| All C_1_S_2_ | | 2.0 | | | | | 70.2 | | | 229.9 | | | | |  | | 6.1 | | | | | | | | | 64.8 | | | | | 256.8 | | | | | |  |  |  |
| Best C_1_S_2_ | | 2.6 | | | | | 70.5 | | | 229.5 | | | | |  | | 6.7 | | | | | | | | | 64.9 | | | | | 260.9 | | | | | |  |  |  |
| Pedigree | | 2.2 | | | | | 69.6 | | | 238.4 | | | | |  | | 6.0 | | | | | | | | | 64.0 | | | | | 259.0 | | | | | |  |  |  |
| Check | | 1.7 | | | | | 69.1 | | | 217.0 | | | | |  | | 4.9 | | | | | | | | | 64.3 | | | | | 249.6 | | | | | |  |  |  |
| Parents | | 1.7 | | | | | 69.0 | | | 221.0 | | | | |  | | 5.3 | | | | | | | | | 63.4 | | | | | 239.6 | | | | | |  |  |  |
|  | |  | | | | | | | | | | | | | | | | | | | | | | | | | | | | | | | | | | |  |  |  |
|  | | --------------------------Population 1019 ------------------- | | | | | | | | | | | | | | | | | | | | | | | | | | | | | | | | | | |  |  |  |
|  | | ----------------------------- *Probability-P* --------------------------- | | | | | | | | | | | | | | | | | | | | | | | | | | | | | | | | | | |  |  |  |
| All C_1_S_2_ vs Pedigree | | 0.16 | | | | | 0.73 | | | 0.69 | | | | |  | | | | | | | | 0.01 | | | 0.01 | | | | | 0.52 | | | | | |  |  |  |
| All C_1_S_2_ vs Checks | | 0.29 | | | | | <.0001 | | | <.0001 | | | | |  | | | | | | | | <.0001 | | | <.0001 | | | | | <.0001 | | | | | |  |  |  |
| Best C_1_S_2_ vs Pedigree | | <.0001 | | | | | 0.38 | | | 0.05 | | | | |  | | | | | | | | 0.01 | | | 0.00 | | | | | 0.00 | | | | | |  |  |  |
| Best C_1_S_2_ vs Checks | | 0.02 | | | | | <.0001 | | | 0.00 | | | | |  | | | | | | | | 0.79 | | | <.0001 | | | | | <.0001 | | | | | |  |  |  |
|  | | ------------------------------------ Mean ----------------------------------- | | | | | | | | | | | | | | | | | | | | | | | | | | | | | | | | | | |  |  |  |
| All C_1_S_2_ | | 2.7 | | | | | 60.0 | | | 195.3 | | | | |  | | | | | | | | 6.0 | | | 62.0 | | | | | 218.2 | | | | | |  |  |  |
| Best C_1_S_2_ | | 3.4 | | | | | 59.8 | | | 202.4 | | | | |  | | | | | | | | 6.9 | | | 62.4 | | | | | 223.8 | | | | | |  |  |  |
| Pedigree | | 2.7 | | | | | 60.1 | | | 195.0 | | | | |  | | | | | | | | 6.5 | | | 61.7 | | | | | 217.9 | | | | | |  |  |  |
| Check | | 3.0 | | | | | 62.9 | | | 212.4 | | | | |  | | | | | | | | 6.9 | | | 64.9 | | | | | 243.1 | | | | | |  |  |  |
|  | | ----------- Drought -stress -------- | | | | | | | | | | | | | | | | |  | | -----------Well-watered ------ | | | | | | | | | | | | | | | | | |  |
|  | | ------------------------Population 1020 -------------------------- | | | | | | | | | | | | | | | | | | | | | | | | | | | | | | | | | | | | |  |
|  | | -------------------------------- *Probability-P* -------------------------------- | | | | | | | | | | | | | | | | | | | | | | | | | | | | | | | | | | | | |  |
| All C_1_S_2_ vs Pedigree | | 0.74 | | | | | 0.02 | | | | 0.12 | | | | | | | |  | | 0.84 | | | | | | <.0001 | | | | | 0.00 | | | | | | |  |
| All C_1_S_2_ vs Checks | | 0.00 | | | | | <.0001 | | | | 0.01 | | | | | | | |  | | 0.16 | | | | | | <.0001 | | | | | <.0001 | | | | | | |  |
| All C_1_S_2_ vs Parents | | 0.16 | | | | | 0.56 | | | | 0.21 | | | | | | | |  | | 0.45 | | | | | | 0.01 | | | | | 0.99 | | | | | | |  |
| Best C_1_S_2_ vs Pedigree | | 0.00 | | | | | 0.00 | | | | 0.32 | | | | | | | |  | | 0.01 | | | | | | <.0001 | | | | | 0.43 | | | | | | |  |
| Best C_1_S_2_ vs Checks | | <.0001 | | | | | <.0001 | | | | 0.07 | | | | | | | |  | | 0.00 | | | | | | <.0001 | | | | | <.0001 | | | | | | |  |
| Best C_1_S_2_ vs Parents | | 0.00 | | | | | 0.14 | | | | 0.17 | | | | | | | |  | | 0.01 | | | | | | 0.00 | | | | | 0.11 | | | | | | |  |
|  | | ------------------------------------- Mean ---------------------------------------- | | | | | | | | | | | | | | | | | | | | | | | | | | | | | | | | | | | | |  |
| All C_1_S_2_ | | 2.7 | | | | | 62.7 | | | | 195.8 | | | | | | | |  | | 5.7 | | | | | | 65.3 | | | | | | | 221.3 | | | | |  |
| Best C_1_S_2_ | | 3.4 | | | | | 62.1 | | | | 197.8 | | | | | | | |  | | 6.1 | | | | | | 65.7 | | | | | | | 227.5 | | | | |  |
| Pedigree | | 2.8 | | | | | 63.2 | | | | 201.5 | | | | | | | |  | | 5.9 | | | | | | 66.5 | | | | | | | 229.5 | | | | |  |
| Check | | 2.6 | | | | | 63.7 | | | | 206.1 | | | | | | | |  | | 5.7 | | | | | | 67.7 | | | | | | | 238.5 | | | | |  |
| Parents | | 2.6 | | | | | 62.5 | | | | 192.4 | | | | | | | |  | | 5.6 | | | | | | 64.7 | | | | | | | 220.9 | | | | |  |
|  | |  | | | | | | | | | | | | | | | | | | | | | | | | | | | | | | | | | | | | |  |
|  | | ------------------------Population 1021-------------------------- | | | | | | | | | | | | | | | | | | | | | | | | | | | | | | | | | | | | |  |
|  | | --------------------------------- *Probability-P* ------------------------------- | | | | | | | | | | | | | | | | | | | | | | | | | | | | | | | | | | | | |  |
| All C_1_S_2_ vs Pedigree | | 0.61 | | | | | 0.35 | | | | 0.27 | | | | | | | |  | | 0.18 | | | | | | 0.89 | | | | | | | <.0001 | | | | |  |
| All C_1_S_2_ vs Checks | | 0.23 | | | | | <.0001 | | | | <.0001 | | | | | | | |  | | 0.33 | | | | | | <.0001 | | | | | | | <.0001 | | | | |  |
| All C_1_S_2_ vs Parents | | 0.53 | | | | | 0.00 | | | | 0.06 | | | | | | | |  | | 0.16 | | | | | | <.0001 | | | | | | | 0.07 | | | | |  |
| Best C_1_S_2_ vs Pedigree | | 0.01 | | | | | 0.44 | | | | 0.17 | | | | | | | |  | | <.0001 | | | | | | 0.35 | | | | | | | <.0001 | | | | |  |
| Best C_1_S_2_ vs Checks | | 0.02 | | | | | 0.00 | | | | <.0001 | | | | | | | |  | | <.0001 | | | | | | <.0001 | | | | | | | <.0001 | | | | |  |
| Best C_1_S_2_ vs Parents | | 0.25 | | | | | 0.01 | | | | 0.03 | | | | | | | |  | | 0.00 | | | | | | <.0001 | | | | | | | 0.02 | | | | |  |
|  | | ------------------------------------- Mean ---------------------------------------- | | | | | | | | | | | | | | | | | | | | | | | | | | | | | | | | | | | | |  |
| All C_1_S_2_ | | 2.6 | | | | | 68.2 | | | | 196.3 | | | | | | | |  | | 6.6 | | | | | | 65.4 | | | | | | | 221.8 | | | | |  |
| Best C_1_S_2_ | | 3.1 | | | | | 68.2 | | | | 195.1 | | | | | | | |  | | 7.2 | | | | | | 65.6 | | | | | | | 224.1 | | | | |  |
| Pedigree | | 2.7 | | | | | 67.7 | | | | 195.0 | | | | | | | |  | | 6.6 | | | | | | 65.4 | | | | | | | 213.1 | | | | |  |
| Check | | 2.8 | | | | | 69.5 | | | | 211.2 | | | | | | | |  | | 6.6 | | | | | | 66.8 | | | | | | | 239.6 | | | | |  |
| Parents | | 2.8 | | | | | 66.0 | | | | 191.8 | | | | | | | |  | | 6.5 | | | | | | 64.0 | | | | | | | 214.8 | | | | |  |
|  | | -----------Drought-stress ------- | | | | | | | | | | | | | | | | |  | | -----------Well-watered ------ | | | | | | | | | | | | | | | | | |  |
|  | | -------------------------Population 1023 -------------------------- | | | | | | | | | | | | | | | | | | | | | | | | | | | | | | | | | | | | |  |
|  | | -------------------------------- Probability-*F* -------------------------------- | | | | | | | | | | | | | | | | | | | | | | | | | | | | | | | | | | | | |  |
| All C_1_S_2_ vs Pedigree | | 0.08 | | | | | 0.06 | | | | <.0001 | | | | | | | |  | | 0.10 | | | | | | <.0001 | | | | | <.0001 | | | | | | |  |
| All C_1_S_2_ vs Checks | | 0.02 | | | | | <.0001 | | | | 0.01 | | | | | | | |  | | <.0001 | | | | | | <.0001 | | | | | <.0001 | | | | | | |  |
| All C_1_S_2_ vs Parents | | 0.91 | | | | | 0.17 | | | | 0.11 | | | | | | | |  | | 0.24 | | | | | | 0.82 | | | | | 0.01 | | | | | | |  |
| Best C_1_S_2_ vs Pedigree | | <.0001 | | | | | 0.15 | | | | <.0001 | | | | | | | |  | | <.0001 | | | | | | <.0001 | | | | | <.0001 | | | | | | |  |
| Best C_1_S_2_ vs Checks | | <.0001 | | | | | <.0001 | | | | 0.00 | | | | | | | |  | | <.0001 | | | | | | <.0001 | | | | | <.0001 | | | | | | |  |
| Best C_1_S_2_ vs Parents | | 0.05 | | | | | 0.21 | | | | 0.36 | | | | | | | |  | | 0.01 | | | | | | 0.64 | | | | | <.0001 | | | | | | |  |
|  | | ------------------------------------- Mean ---------------------------------------- | | | | | | | | | | | | | | | | | | | | | | | | | | | | | | | | | | | | |  |
| All C_1_S_2_ | | 2.1 | | | | | 70.8 | | | | 211.2 | | | | | | | |  | | 6.8 | | | | | | 68.3 | | | | | | | 234.2 | | | | |  |
| Best C_1_S_2_ | | 2.8 | | | | | 70.9 | | | | 215.5 | | | | | | | |  | | 7.3 | | | | | | 68.4 | | | | | | | 240.8 | | | | |  |
| Pedigree | | 1.9 | | | | | 71.3 | | | | 200.6 | | | | | | | |  | | 6.7 | | | | | | 69.4 | | | | | | | 228.2 | | | | |  |
| Check | | 1.9 | | | | | 69.5 | | | | 203.3 | | | | | | | |  | | 5.8 | | | | | | 66.2 | | | | | | | 228.0 | | | | |  |
| Parents | | 2.2 | | | | | 71.5 | | | | 217.0 | | | | | | | |  | | 6.4 | | | | | | 68.2 | | | | | | | 222.8 | | | | |  |
|  | |  | | | | | | | | | | | | | | | | | | | | | | | | | | | | | | | | | | | | |  |
|  | | ---------------------------Population 1028 ----------------------- | | | | | | | | | | | | | | | | | | | | | | | | | | | | | | | | | | | | |  |
|  | | --------------------------------- *Probability-P* ------------------------------- | | | | | | | | | | | | | | | | | | | | | | | | | | | | | | | | | | | | |  |
| All C_1_S_2_ vs Pedigree | | 0.29 | | | | | 0.90 | | | | 0.37 | | | | | | | |  | | 0.20 | | | | | | 0.06 | | | | | | | <.0001 | | | | |  |
| All C_1_S_2_ vs Checks | | 0.13 | | | | | 0.01 | | | | 0.00 | | | | | | | |  | | <.0001 | | | | | | <.0001 | | | | | | | <.0001 | | | | |  |
| All C_1_S_2_ vs Parents | | 0.03 | | | | | 0.47 | | | | 0.21 | | | | | | | |  | | 0.25 | | | | | | 0.00 | | | | | | | <.0001 | | | | |  |
| Best C_1_S_2_ vs Pedigree | | <.0001 | | | | | 0.63 | | | | 0.02 | | | | | | | |  | | <.0001 | | | | | | 0.05 | | | | | | | <.0001 | | | | |  |
| Best C_1_S_2_ vs Checks | | <.0001 | | | | | 0.01 | | | | <.0001 | | | | | | | |  | | <.0001 | | | | | | <.0001 | | | | | | | <.0001 | | | | |  |
| Best C_1_S_2_ vs Parents | | 0.00 | | | | | 0.37 | | | | 0.05 | | | | | | | |  | | 0.00 | | | | | | 0.00 | | | | | | | <.0001 | | | | |  |
|  | | ------------------------------------- Mean ---------------------------------------- | | | | | | | | | | | | | | | | | | | | | | | | | | | | | | | | | | | | |  |
| All C_1_S_2_ | | 2.6 | | | | | 71.4 | | | | 220.6 | | | | | | | |  | | 6.7 | | | | | | 70.2 | | | | | | | 237.3 | | | | |  |
| Best C_1_S_2_ | | 3.3 | | | | | 71.8 | | | | 228.6 | | | | | | | |  | | 7.3 | | | | | | 70.4 | | | | | | | 241.5 | | | | |  |
| Pedigree | | 2.6 | | | | | 71.3 | | | | 225.5 | | | | | | | |  | | 6.7 | | | | | | 69.8 | | | | | | | 227.0 | | | | |  |
| Check | | 2.6 | | | | | 68.9 | | | | 212.9 | | | | | | | |  | | 6.2 | | | | | | 66.8 | | | | | | | 225.6 | | | | |  |
| Parents | | 2.1 | | | | | 70.0 | | | | 219.3 | | | | | | | |  | | 6.5 | | | | | | 68.5 | | | | | | | 219.2 | | | | |  |
